# HIV-Neutralizing Activity of Cationic Polypeptides in Cervicovaginal Secretions of Women in HIV-Serodiscordant Relationships

**DOI:** 10.1371/journal.pone.0031996

**Published:** 2012-02-28

**Authors:** Pauline Levinson, Robert Y. Choi, Amy L. Cole, Taha Hirbod, Samuel Rhedin, Barbara Payne, Brandon L. Guthrie, Rose Bosire, Alexander M. Cole, Carey Farquhar, Kristina Broliden

**Affiliations:** 1 Unit of Infectious Diseases, Department of Medicine, Solna, Center for Molecular Medicine, Karolinska Institutet, Karolinska University Hospital, Stockholm, Sweden; 2 Department of Paediatrics and Child Health, University of Nairobi, Nairobi, Kenya; 3 Department of Medicine, University of Washington, Seattle, Washington, United States of America; 4 Department of Molecular Biology and Microbiology, Burnett School of Biomedical Sciences, University of Central Florida College of Medicine, Orlando, Florida, United States of America; 5 Department of Global Health, University of Washington, Seattle, Washington, United States of America; 6 Department of Epidemiology, University of Washington, Seattle, Washington, United States of America; 7 Centre for Public Health Research, Kenya Medical Research Institute, Nairobi, Kenya; University of California San Francisco, United States of America

## Abstract

**Background:**

HIV exposed seronegative (HESN) women represent the population most in need of a prophylactic antiviral strategy. Mucosal cationic polypeptides can potentially be regulated for this purpose and we here aimed to determine their endogenous expression and HIV neutralizing activity in genital secretions of women at risk of HIV infection.

**Methodology/Principal Findings:**

Cervicovaginal secretions (CVS) of Kenyan women in HIV-serodiscordant relationships (HESN, n = 164; HIV seropositive, n = 60) and low-risk controls (n = 72) were assessed for the cationic polypeptides HNP1–3, LL-37 and SLPI by ELISA and for HIV neutralizing activity by a PBMC-based assay using an HIV primary isolate. Median levels of HNP1–3 and LL-37 in CVS were similar across study groups. Neither HSV-2 serostatus, nor presence of bacterial vaginosis, correlated with levels of HNP1–3 or LL-37 in the HESN women. However, an association with their partner's viral load was observed. High viral load (>10,000 HIV RNA copies/ml plasma) correlated with higher levels of HNP1–3 and LL-37 (p = 0.04 and 0.03, respectively). SLPI was most abundant in the low-risk group and did not correlate with male partner's viral load in the HESN women. HIV neutralizing activity was found in CVS of all study groups. In experimental studies, selective depletion of cationic polypeptides from CVS rendered the remaining CVS fraction non-neutralizing, whereas the cationic polypeptide fraction retained the activity. Furthermore, recombinant HNP1–3 and LL-37 could induce neutralizing activity when added to CVS lacking intrinsic activity.

**Conclusions/Significance:**

These findings show that CVS from HESN, low-risk, and HIV seropositive women contain HIV neutralizing activity. Although several innate immune proteins, including HNP1–3 and LL-37, contribute to this activity these molecules can also have inflammatory properties. This balance is influenced by hormonal and environmental factors and in the present HIV serodiscordant couple cohort study we show that a partner's viral load is associated with levels of such molecules.

## Introduction

Mucosal surfaces of the female genital tract are portals for sexual transmission of microbes and viruses, and cationic antimicrobial polypeptides are the principal effector molecules of mucosal innate immunity. Together with other innate immune cells and molecules, as well as physical and chemical barriers (mucus, low pH, epithelial integrity), these antimicrobials normally contribute to an efficient barrier against low dose HIV exposure during sexual intercourse with an HIV infected partner [1], [2]. Innate immune molecules may thus contribute to the natural immune defense against HIV in genital tract secretions. Prior innate immunity studies focused on HIV-infected and uninfected women from low HIV-endemic regions [3], [4], [5] and highly HIV exposed female sex workers [6], [7], [8], . Antiviral cationic polypeptides are secreted from immune and epithelial cells into cervicovaginal secretions (CVS) and can potentially be regulated selectively by vaccines and microbicides against mucosal infections. The desired modulation of innate immune responses must, however, be balanced to avoid stimulation of immunopathological effects.

Several cationic polypeptides such as human neutrophil peptides 1–3 (HNP1–3), LL-37 and secretory leukocyte protease inhibitor (SLPI) have been shown to possess HIV inhibitory activity *in vitro* and are present at detectable levels in CVS (reviewed by [11]). HNP1–3 are members of the human alpha-defensin family and inhibit HIV replication through intracellular interference with protein kinase C activity and direct inactivation of HIV virions [12], [13]. LL-37 belongs to the family of cathelicidins and mediates HIV inhibitory activity [14], although the antiviral mechanism is not clear. SLPI is a serine protease inhibitor that exerts one of its anti-HIV effects by binding to annexin II and impairing annexin II-mediated stabilization of fusion [15], [16].

Each of these synergistically acting molecules can have multifaceted roles influenced by the immunologic, hormonal, and microbial status of the genital mucosal surface of sexually active individuals. In a previous study, we showed that cationic polypeptides were required for anti-HIV activity of human vaginal fluid from healthy women at low risk of sexual HIV exposure [5]. We now extend these studies and explore the anti-HIV activity of human CVS from Kenyan HIV infected and uninfected women with differing risks of HIV infection. The HIV exposed seronegative (HESN) women, who represent the population most in need of a prophylactic vaccine or microbicide compound, have a high prevalence of herpes simplex virus type 2 (HSV-2), bacterial vaginosis (BV) and other factors influencing the mucosal environment and innate immune composition.

## Materials and Methods

### Study population

During the years 2007–2010, HIV serodiscordant (n = 469) couples were enrolled in a prospective cohort study in Nairobi, Kenya and followed for up to 2 years with visits every 3 months [17]. Eligible couples reported sexual intercourse with each other ≥3 times in the 3 months prior to screening, planned to remain together in Nairobi for the two-year duration of the study, female partners could not be pregnant at enrollment, and the HIV-positive partners did not have clinical AIDS (WHO stage IV) or a history of taking antiretroviral treatment. Additionally, for the present sub-study, HIV seropositive and HIV seronegative women were selected from this larger cohort based on sample availability. Women were excluded if they showed evidence of an active sexually transmitted infection (STI) on genital examination. During the same time period, couples testing concordantly negative for HIV were recruited from the same voluntary counseling and testing (VCT) centers as the discordant couples as a control population. Concordant negative couples also reported having sex with their partner ≥3 times in the 3 months prior to screening. A subset of women from these negative control couples was selected based on sample availability and assigned to the ‘Low-risk’ study group. The study protocols were approved by institutional review boards at the Kenyatta National Hospital (Nairobi, Kenya), the University of Washington (Seattle, USA) and the Karolinska Institutet (Stockholm, Sweden). Written consent was obtained from all study participants.

### Sample collection

For the present study, CVS were collected from these women at the time of study enrollment and a questionnaire was administered by a nurse-counselor that included sociodemographic data and sexual/reproductive history. A physical examination, including a genital examination, was performed. CVS were collected by rotating a cotton swab 360° in the outer part of the endocervix and by rotating a different swab across the vaginal wall. Both swabs were placed in the same tube containing 5 ml sterile phosphate buffered saline (PBS), which was immediately placed on ice. The samples were then transported from the clinic to the laboratory on ice and centrifuged at 800 g for 10 minutes at 4°C. Supernatants were separated from the cell pellet, aliquoted into 4 cryovials, each containing 1 ml fluid, and stored at −80°C. CVS from healthy Swedish low-risk HIV seronegative women were collected and stored according to the same procedure and were used as internal controls for the *in vitro* experiments.

### Study subjects and sample collection for the *in vitro* experiments

For cationic polypeptide depletion experiments, a more concentrated CVS sample was required. HIV seronegative women, without any clinical or laboratory signs of STIs (chlamydia, gonorrhea, syphilis, or candida) or BV, were recruited from the Maternal and Child Health Clinic in Pumwani, Nairobi. One cotton swab soaked in mucus from the cervico-vaginal tract was placed in 0.5 ml of saline and placed on ice. The vials were transported to the laboratory where the samples were stored in −80°C.

### HIV testing and STI screening

HIV rapid testing was conducted in the Study Clinic using two commercial kits (Determine**®** HIV–1/2 Rapid Test, Abbott Laboratories, Abbott Park, IL; Bioline™ Recombigen HIV Test, Standard Diagnostic Inc.). Positive or indeterminate results were confirmed with HIV-1 enzyme-linked immunosorbent assay (ELISA) using the Vironostika® HIV Uni-Form II Ag/Ab ELISA kit (Biomerioux Laboratories, the Netherlands) in the University of Nairobi's Obstetrics/Gynecology laboratory. Plasma HIV-1 RNA viral load in HIV-seropositive partners was quantified using the Gen-Probe Transcription Mediated Amplification (TMA) assay [18]. HSV-2 serostatus was determined using the HerpeSelect IgG ELISA kit (Focus Technologies, Cyprus, CA) using a cutoff of 3.5 to improve specificity. Syphilis testing was performed in University of Nairobi's Medical Microbiology Laboratory using the rapid plasma regain (RPR) Card Test Macro-Vue kit (Becton Dickinson, Franklin Lakes, NJ), with reactive RPR tests confirmed with TPHA, a treponemal-specific indirect hemagglutination test (RANDOX Laboratories, Crumlin, UK). Vaginal Gram test was performed and BV was defined as a Nugent score of 7–10.

### Measurement of prostate-specific antigen (PSA)

Prostate-specific antigen (PSA) levels in CVS were measured by an external accredited laboratory (Aleris Medilab, Täby, Sweden) using the ARCHITECT Total PSA assay (Abbott Ireland Diagnostics Division, Dublin, Ireland), a chemiluminescent microparticle immunoassay. The PSA levels in the CVS are reported as measured in the original 5 ml dilution. A level above 1 µg PSA/mL CVS were defined as a positive result [19].

### HNP1–3, LL-37 and SLPI quantification

Commercial ELISA kits were used, according to the manufacturers protocols, to quantify the immune molecules of interest at the following dilutions: the α-defensins HNP-1–3 (1∶500), LL-37 (1∶10) (both Hycult Biotechnology, Uden, the Netherlands), and SLPI (1∶100-1∶1000) (RD Systems Europe Ltd, Abingdon Oxon, UK). The CVS levels of the factors are reported as measured in the original 5 ml dilution.

### HIV neutralization assay

HIV neutralization assays were performed according to a predefined protocol and neutralization cut off [20]. Prior to this assay, the IgA1 fraction of the CVS was removed by adding 800 µl of undiluted CVS to 400 µl of jacalin-agarose beads (Immunkemi, Stockholm, Sweden) then mixing for 2 h at +4°C followed by centrifugation (2000 rpm, 5 min, 4°C). The unbound (IgA1-depleted) fraction was collected and stored at −80°C until use. For the HIV neutralizing assay we used an R5 tropic primary isolate of HIV subtype A (isolate 92UG037; AIDS Research and Reference Reagent Program, Division of AIDS, NIAID, NIH). To account for variations in TCID_50_ depending on PBMC donor variability, three viral dilutions were used in each assay. Duplicate wells of 75 µl of each virus dilution and 75 µl of each sample fraction (undiluted) were incubated for 1 h at 37°C followed by addition of a mixture of 1×10^5^ PHA-P-stimulated PBMC from two donors. After 24 h incubation at 37°C, the cells were centrifuged; unbound virus was washed away, and 200 µl of fresh medium were added to each well. On day 3, 120 µl of medium was discarded and replaced with new medium, after which day 6 supernatants were collected for analysis of virus production with a p24 antigen ELISA (Vironostika HIV-1 Antigen; Electra-Box Diagnostica AB, Stockholm, Sweden). Neutralization was defined as a ≥67% reduction of p24 antigen in the supernatant as compared with p24 antigen content when the virus isolate was incubated in the presence of a standard pool of Swedish HIV seronegative samples.

### Selective depletion of cationic polypeptides from CVS

Carboxymethyl (CM) weak cation exchange resin (Bio-Rad) was used to deplete cationic polypeptides from vaginal fluid [5]. The CM resin was pre-equilibrated by washing 6 times with a buffer resembling vaginal fluid in electrolyte composition (60 mM NaCl, 20 mM potassium phosphate, pH 6, [4]). Equal volumes of CVS from four to six HIV seronegative donors were centrifuged and pooled before the extraction of cationic polypeptides. From each pool, 0.5 mL CVS was reserved and stored at −80°C as “unprocessed CVS”. The remaining volume from each pool was CM-extracted by mixing with equilibrated CM resin at 4°C overnight in an end-over-end tumbler. Centrifugation (15,000×g, 4°C, 3 min) enabled collection of the CM-depleted CVS supernatant, which was then cleared of residual resin by additional centrifugations and stored at −80°C. The CM resin sediment was washed 5 times with 25 mM ammonium acetate, pH7, and then cationic proteins were extracted by incubating the washed resin with 5% acetic acid in a tumbler at 4°C. Extracted proteins were collected and stored at −80°C after 2 h, and the resin was extracted a second time by incubation with more acetic acid overnight in a tumbler at 4°C. The first and second cationic extracts were pooled, clarified of residual resin, concentrated by vacuum centrifugation, and restored to the original volume. All samples were stored at −80°C until use.

### Recombinant innate factors and HIV neutralization

Recombinant SLPI (RD Systems Europe Ltd, Abingdon Oxon, UK), LL37 and HNP1–3 (both Hycult Biotechnology, Uden, the Netherlands) were added in serial dilutions to IgA-depleted CVS samples from low-risk HIV seronegative women who were selected based on lack of intrinsic neutralizing capacity. The recombinant peptides were incubated with the CVS samples for 1 hr. Following this pre-incubation, the samples were tested in the HIV neutralization assay as described above except for the use of another HIV isolate of the same HIV subtype (subtype A: RW/92/024; AIDS Research and Reference Reagent Program, Division of AIDS, NIAID, NIH).

### Statistical analysis

Statistical comparisons of the immune parameters between groups were performed by using the Mann-Whitney U test, and calculations were performed by using the GraphPad Prism 5 software (GraphPad Software, Inc., San Diego, CA, USA) and STATA version 11.2/IC (College Station, TX, USA). A p-value of <0.05 was considered statistically significant.

Owing to lack of sufficient volume of some CVS samples, not all 300 samples were assessed for all parameters in the following sections. However, the number of samples assessed in each case is presented in the table and figure legends.

Because a partner's viral load of 10,000 or greater has been shown as an epidemiologic determinant for higher transmission risk in this geographical setting [21], this content was selected as a cut-off for dividing the HESN women into two groups: HESN women whose partner's viral load was higher than 10,000 versus HESN women whose partner's viral load was lower than 10,000.

## Results

### Demographic data and levels of antiviral cationic polypeptides in CVS samples

In the present study, CVS samples were assessed from a total of 296 study participants: HIV seropositive (HIV pos, n = 60) and HIV seronegative (HESN, n = 164) women representing the serodiscordant couple cohort as well as HIV seronegative (Low-risk, n = 72) women living with a seroconcordant HIV-negative male partner. Demographic data and sexual risk-taking profiles of the study subjects are outlined in Table 1. While number of life time partners, history of STIs, presence of BV and male partner circumcision status were comparable, the parameters age, presence of PSA, and HSV-2 seropositivity differed between the groups.

**Table 1:** Enrollment characteristics of study population at date of sample collection.

	HIV-pos^a^ (n = 60)	HESN^b^ (n = 164)	Low-risk^c^ (n = 72)
*Characteristics of female study subjects*	Median or number (interquartile range or %)		
Age (years)	28 (24–31)	29 (25–35)	25 (22–30)
Lifetime partners	3 (2–4)	3 (2–3)	3 (2–3)
History of STIs^d^	18 (33%)	34 (22%)	17 (24%)
Presence of PSA^e^	5 (12%)	15 (10%)	23 (33%)
Presence of BV	6 (10%)	22 (14%)	14 (25%)
HSV-2 seropositivity	32 (53%)	111 (68%)	22 (33%)
*Characteristics of male partners*			
Age (years)	32 (28–36)	36 (32–43)	
Lifetime partners	5 (3–10)	5 (4–9)	
History of STIs^d^	18 (35%)	77 (48%)	
Circumcision	46 (78%)	116 (71%)	
HSV-2 seropositivity	20 (33%)	109 (66%)	
CD4 cell count (cells per ul/100)		355 (237–517)	
Viral load (log10 copies/ml)		4.76 (4.07–5.34)	
*Characteristics of couples*			
Sex act with study partner in last 6 months	6 (3–10)	4.5 (2–8)	
Years living together	3 (2–6)	6 (2–10)	

a–c HIV seropositive (HIV pos) and HIV seronegative (HESN) women and their HIV serodiscordant male partners were evaluated for demographic data and sexual risk-taking profiles. In addition, HIV seronegative women (Low-risk) living with an HIV seronegative male partner were included (male participants in the Low-risk study group were not assessed for demographic parameters). In total, complete demographic data sets were available from 288 of the 300 women whose CVS samples were assessed for immunological parameters.

d STIs: sexually transmitted infections.

e PSA: Prostate-specific antigen (>1 ug/ml of CVS) as a sign of recent unprotected sexual intercourse.

**Figure 1:**
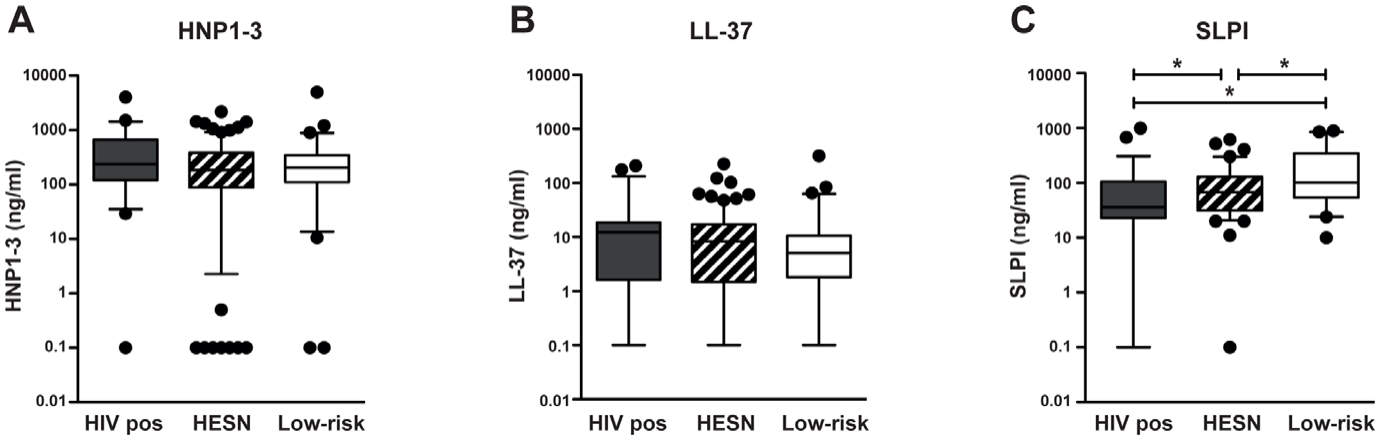
Levels of cationic polypeptides in the study groups. CVS were collected from HIV seropositive women (HIV pos) as well as from HIV seronegative women living with either an HIV infected man (HESN) or an HIV uninfected man (Low-risk). Levels of the respective cationic polypeptide for the different study groups are shown. (a) HNP1–3 (HIV-pos *n = 52*, HESN *n = 163,* low-risk *n = 64*), (b) LL-37 (HIV-pos *n = 52*, HESN *n = 162,* low-risk *n = 63*) and (c) SLPI (HIV-pos *n = 60*, HESN *n = 86,* low-risk *n = 41*). Assay detection level was 0.16 ng/ml for HNP1–3, 0.14 for LL-37 and 0.06 for SLPI, results below cut-off were assigned the value y = 0,1. Solid line indicates median concentration, whiskers indicate 5–95 percentile. *p<0.05.

The most abundant peptide among the ones investigated (HNP1–3, LL-37, SLPI) in all three study groups was HNP1–3, with comparable median levels for the HIV-positive, HESN and low-risk groups (median values 240, 185 and 205 ng/ml, respectively) (Figure 1). Also, levels of LL-37 were comparable between the groups (median values 12, 8 and 5 ng/ml, respectively). However, less SLPI was present in the HIV positive group than in the other two groups (median values 37, 64 and 106 ng/ml, respectively) (HIV pos vs HESN: p = 0.013; HIV pos vs Low-risk: p<0.001); likewise, the HESN group contained less SLPI than the low-risk group (HESN vs Low-risk p = 0.005) (Figure 1).

### Levels of cationic polypeptides in relation to partner's viral load, HSV-2 serostatus, and presence of BV

**Figure 2:**
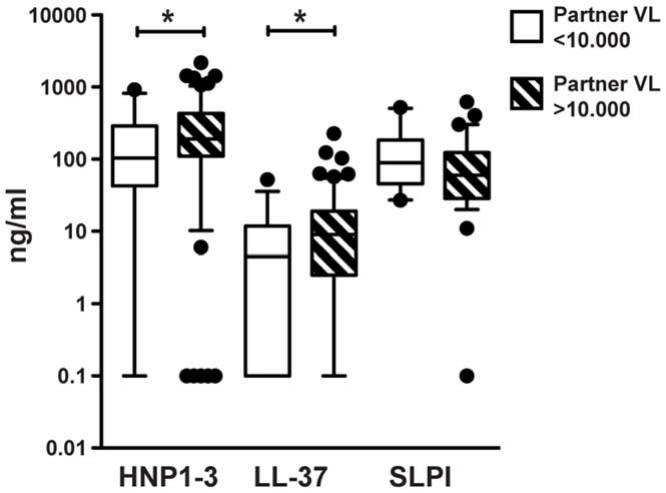
Correlations of partner's viral load with levels of cationic polypeptides in the HESN group of women. Expression of cationic polypeptides in CVS from HIV seronegative women in a serodiscordant relationship (the HESN group) was stratified for partner's viral load (VL, HIV RNA copies/ml plasma) at time of sample collection. ELISA-data of levels of HNP1–3 (VL<10.000 *n = 37,* VL>10.000 *n = 126),* LL-37 (VL<10.000 *n = 37,* VL>10.000 *n = 125*) and SLPI (VL<10.000 *n = 20,* VL>10.000 *n = 66*). Assay detection level was 0.16 ng/ml for HNP1–3, 0.14 for LL-37 and 0.06 for SLPI, results below cut-off were assigned the value y = 0.1. Solid line indicates median concentration, whiskers indicate 5–95 percentile. *p<0.05.

We examined several mucosal factors that could potentially influence the levels of cationic polypeptides. First, in the HESN group, partner's viral load (greater than or less than 10,000 HIV RNA copies/ml plasma) was compared to levels of the individual peptides (Figure 2). HESN women whose partner's viral load was higher than 10,000 (n = 127) had significantly higher levels of HNP1–3 and LL37 than HESN women whose partner's viral load was less than 10,000 (n = 37) (HNP1–3: 191 vs 109 ng/ml, p = 0.036; LL-37: 9 vs 4 ng/ml, p = 0.028). Levels of SLPI were however not affected by high or low levels of partner's viral load.

**Figure 3:**
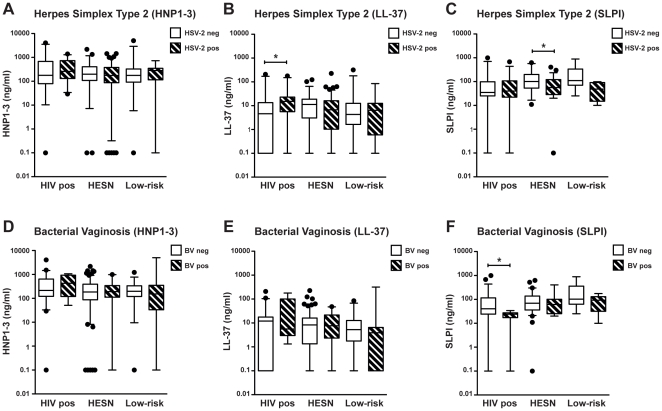
Correlations of HSV-2 serostatus and presence of BV with levels of cationic polypeptides in the study groups. CVS were collected from HIV seropositive women (HIV pos) as well as from HIV seronegative women living with either an HIV infected man (HESN) or an HIV uninfected man (Low-risk). Comparison of women with or without HSV-2 infection (HSV+/−): Levels of (a) HNP1–3 (HIV− pos HSV− *n = 23*, HIV-pos HSV+ *n = 29*, HESN HSV− *n = 52*, HESN HSV+ *n = 110*, low-risk HSV− *n = 37*, low-risk HSV+ *n = 22*), (b) LL-37 (HIV-pos HSV− *n = 24*, HIV-pos HSV+ *n = 28*, HESN HSV− *n = 52*, HESN HSV+ *n = 109*, low-risk HSV− *n = 36*, low-risk HSV+ *n = 22*) and (c) SLPI (HIV-pos HSV− *n = 28*, HIV-pos HSV+ *n = 32*, HESN HSV− *n = 28*, HESN HSV+ *n = 58*, low-risk HSV− *n = 28*, low-risk HSV+ *n = 9*). Comparison of women with or without BV (BV+/−): (d) Expression of HNP1–3 (HIV-pos BV− *n = 47*, HIVpos BV+ *n = 4*, HESN BV− *n = 138*, HESN BV+ *n = 22*, low-risk BV− *n = 38*, low-risk BV+ *n = 11*). (e) Expression of LL-37 (HIV-pos BV− *n = 46*, HIV-pos BV+ *n = 5*, HESN BV− *n = 137*, HESN BV+ *n = 22*, low-risk BV− *n = 38*, low-risk BV+ *n = 11*). (f) Expression of SLPI (HIV-pos BV− *n = 53*, HIV-pos BV+ *n = 6*, HESN BV− *n = 71*, HESN BV+ *n = 14*, low-risk BV− *n = 19*, low-risk BV+ *n = 8*). Assay detection level was 0.16 ng/ml for HNP1–3, 0.14 for LL-37 and 0.06 for SLPI, results below cut-off were assigned the value y = 0.1. Solid line indicates median concentration, whiskers indicate 5–95 percentile. *p<0.05.

We next assessed the association of HSV-2 serostatus and presence of BV with levels of the peptides in all study groups. HIV positive women who were co-infected with HSV-2 had significantly higher levels of LL-37 than those who were HSV-2 seronegative (16 vs 5 ng/ml, p = 0.016). Among the HESNs, lower levels of SLPI were observed for the HSV-2 seropositive as compared with the HSV-2 seronegative women (55 vs 99 ng/ml, p = 0.030) (Figure 3a–c). For HESN and low-risk controls, no significant differences were seen when comparing the influence of BV on peptide levels. Presence of BV was however associated with higher levels of SLPI in HIV positive women (median levels of SLPI in BV positive vs BV negative women: 42 vs 24 ng/ml, p = 0.035) (Figure 3d–f).

### Levels of cationic polypeptides in relation to presence or absence of PSA in the CVS samples

To evaluate the influence of seminal contamination in the CVS, samples were assessed for presence of PSA (>1 µg/ml) and then compared for levels of cationic polypeptides. PSA was found in 33% of the CVS samples representing the low-risk group followed by 12% in the HIV positive group and 10% in the HESN group (Table 1). Assessing all study groups together, no statistically significant differences were seen between PSA positive and PSA negative CVS samples for levels of HNP1–3 (median levels: 160 vs 205 ng/ml, p = 0.19). Levels of LL-37 were however significantly lower among the PSA positive versus the PSA negative CVS samples (median levels 3 vs 7 ng/ml, p = 0.015), whereas levels of SLPI were significantly higher among the PSA positive versus the PSA negative CVS samples (median levels: 101 vs 59 ng/ml, p = 0.029). Reanalysing the data presented in Figure 1 by excluding the PSA-positive samples did not affect the median values of the peptide levels significantly (data not shown). However, for LL-37, exclusion of PSA-positive samples resulted in a significant difference between the HIV-positive and low-risk groups (13 vs 5 ng/ml, p = 0.04).

### Levels of cationic peptides in relation to HIV neutralization capacity

As previously shown [22], the IgA fraction of whole CVS could potentially neutralize HIV in our PBMC-based neutralizing assay in a proportion of the present samples. We therefore depleted IgA from whole CVS prior to assessment of neutralizing capacity of the samples to avoid the influence of this factor. In 27 of 152 (18%) IgA-depleted samples from the HESN women, HIV was neutralized. In the low-risk group, the corresponding proportion was 17 of 63 (27%) and in the HIV positive group 17 of 46 (37%). When excluding PSA-positive samples from the analysis the corresponding percentages were 19%, 30% and 30% for the HESN, low-risk and HIV positive group, respectively.

**Figure 4:**
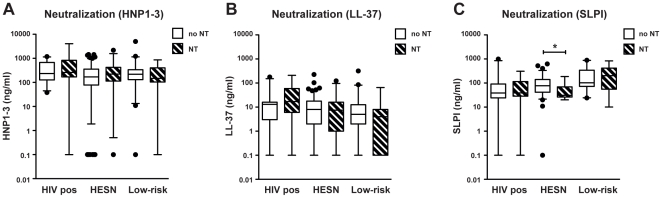
Correlation of HIV neutralizing activity and levels of cationic polypeptides in the study groups. CVS were collected from HIV seropositive women (HIV pos) as well as from HIV seronegative women living with either an HIV infected man (HESN) or an HIV uninfected man (Low-risk). HIV neutralization capacity (NT) of IgA-depleted CVS: The study samples were sorted by whether neutralization occurred (NT) or not (no NT). Levels of (a) HNP1–3 (HIV-pos no NT *n = 26*, HIV-pos NT *n = 15*, HESN no NT *n = 124*, HESN NT *n = 27*, low-risk no NT *n = 43*, low-risk NT *n = 14*), (b) LL-37 (HIV-pos no NT *n = 28*, HIV-pos NT *n = 15*, HESN no NT *n = 123*, HESN NT *n = 27*, low-risk no NT *n = 41*, low-risk NT *n = 15*) and (c) SLPI (HIV-pos no-NT *n = 29*, HIV-pos NT *n = 17*, HESN no NT *n = 73*, HESN NT *n = 12*, low-risk no NT *n = 25*, Ctrl NT *n = 11*). Assay detection level was 0.16 ng/ml for HNP1–3, 0.14 for LL-37 and 0.06 for SLPI, results below cut-off were assigned the value y = 0.1. Solid line indicates median concentration, whiskers indicate 5–95 percentile. *p<0.05.

Samples from the study groups were then divided based on the presence/absence of neutralization capacity and within each study group and comparison was made between neutralizing and non-neutralizing samples in terms of their levels of each peptide (Figure 4). No statistically significant differences were seen between neutralizing and non-neutralizing samples for levels of HNP1–3 or LL-37 in any of the study groups. In the HESN group, samples that could neutralize HIV had significantly lower levels of SLPI (31 ng/ml vs 76 ng/ml; p = 0.02).

### Cationic polypeptides contribute substantially to the HIV-neutralizing activity of CVS from HIV seronegative individuals

**Figure 5:**
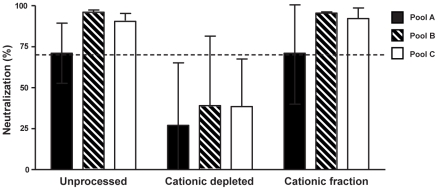
Cationic polypeptides contribute substantially to the HIV-neutralizing activity of CVS from HIV seronegative women. The HIV neutralizing activities of unprocessed CVS, cationic-depleted CVS and cationic polypeptides extracted from the CM resin (the cationic fraction); all were tested individually for each pool of CVS samples. The concentrations of LL37 and HNP 1–3, respectively, in the different pools were: A: 4 and 30 ng/ml, B: 2 and 10 ng/ml, C: 2 and 10 ng/ml. The results shown are from one representative experiment using duplicate wells and two different virus dilutions (median values ±SEM).

In this set of experiments, we explored the HIV-neutralizing activity of the cationic polypeptide components of CVS from HIV seronegative women. A total of 14 CVS samples were selected for the presence of intrinsic HIV neutralizing activity and pooled into three groups to reach sufficient volume for the following experiments (Figure 5). First, the cationic polypeptides were selectively removed from the CVS pools while sparing the concentrations of remaining proteins and electrolytes. The complete removal of cationic polypeptides was confirmed by AU-PAGE for all pools (data not shown). The cationic polypeptide fractions were then tested for HIV neutralizing activity, and each fraction had activity equivalent to that of the whole pool (Figure 5). The remaining peptide-depleted CVS samples had no HIV neutralizing activity. Thus, the majority of the functional activity seems to be mediated by the cationic polypeptide fraction.

### Recombinant cationic polypeptides can augment the intrinsic HIV-neutralizing activity of CVS from HIV low-risk women

**Figure 6:**
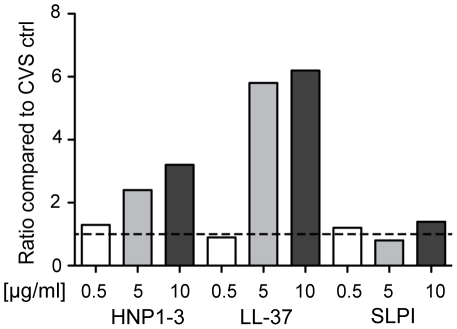
Recombinant cationic polypeptides can augment the intrinsic HIV-neutralizing activity of CVS from HIV seronegative women. The HIV-inhibiting effect of recombinant forms of HNP1–3, LL-37 and SLPI was assessed for HIV neutralizing activity by pre-incubating them individually in serial dilutions with CVS. The CVS samples were selected based on lack of intrinsic neutralizing activity. The ratio of HIV neutralizing activity was calculated as: CVS plus peptide divided by CVS alone. One representative experiment from at least three independent experiments is shown.

Next we studied whether adding individual recombinant cationic polypeptides to CVS could induce HIV neutralizing activity. Theoretically, the added peptides would act synergistically with other innate immune factors naturally present in CVS. For these experiments, CVS from HIV seronegative women lacking intrinsic HIV neutralizing activity were selected and the HIV neutralizing assay was adapted to establish the ratio between peptide+CVS versus medium+CVS. SLPI as well as recombinant HNP1–3 and LL-37 were evaluated in three independent experiments. As a result, both HNP1–3 and LL-37 induced a two to six-fold increase of HIV inhibiting activity when assessed at about 10–50 times the physiological concentrations, whereas the effect of SLPI was only marginal (Figure 6). At physiological concentrations (as determined within the present study cohort), no effect was seen for any of the peptides.

## Discussion

We document here the presence of cationic polypeptides and intrinsic HIV neutralizing activity in CVS of Kenyan women who are HIV infected, HESN, and HIV-uninfected low risk. The three cationic polypeptides HNP1–3, LL-37 and SLPI were selected for assessment because they were previously shown to be important mediators of HIV neutralization *in vitro,* and because they are detectable in cervicovaginal lavage from healthy women (reviewed in [11]). Although presence of HIV neutralizing activity did not associate directly with individual levels of HNP1–3 or LL-37, it was clearly correlated with the whole cationic polypeptide fraction of CVS. This correlation was shown *in vitro* by selectively depleting the cationic polypeptides from CVS, which we demonstrated significantly reduced the functional activity. In another set of experiments, human CVS lacking intrinsic HIV neutralizing activity gained this function following addition of recombinant HNP1–3 or LL-37, but only marginally from SLPI.

Detailed demographic data were collected from the study participants. Despite being thoroughly counseled on sexual risk behavior, signs of recent unprotected sexual intercourse as assessed by measurement of PSA were documented in 10% of the HESN women and in 12% of the HIV seropositive women. The corresponding figure in the low risk women was 33%. Furthermore, none of the HIV infected partners of the HESN women were on ARV treatment at enrollment; about two-thirds of the partners had a plasma viral load above 10,000 HIV RNA copies per ml, a threshold shown to correlate with a high risk of HIV transmission in this setting [21]. When the HIV infected partners were stratified into two groups according to viral load (more or less than 10,000), significant associations were seen with cationic polypeptide levels in the CVS of the corresponding HIV uninfected partner; a partner's viral load above 10,000 associated with higher levels of HNP1–3 and LL-37, whereas no association was seen for SLPI. Thus, although seminal fluid and plasma viral load may not be directly correlated, sexual exposure to a high viral load may have provoked a mucosal inflammatory response, including the release of these peptides into the genital secretions.

Levels of the cationic polypeptides were also related to study group, HSV-2 serostatus and presence of BV. Levels of both HNP1–3 and LL-37 were similar when compared between study groups, whereas SLPI levels were lowest in the HIV positive group and highest in the low risk group. SLPI can be degraded by endogenous proteases and by proteolytic activity of pathogenic agents and, although not assessed here, HIV positive individuals differ in many aspects with regard to the genital mucosal environment that could account for the findings. HIV infected individuals who were HSV-2 seropositive had higher levels of LL-37 than their HSV-2-seronegative counterparts within the same study group. In HESNs, levels of SLPI were lower for HSV-2 seropositive than HSV-2 seronegative individuals. Active HSV-2 infection and bacterial STIs have previously been shown to affect levels of cationic polypeptides, including increased levels of LL-37 and HNP1–3 in Kenyan high risk women [9], [10], [23]. No association between levels of LL-37 and HNP1–3 were seen when the study groups were evaluated for the presence of BV which is in agreement with our previous data [9]. In studies of a low risk group from a low HIV-endemic region, BV was associated with decreased levels of defensins and SLPI [11], [24], and in the present study we noted lower levels of SLPI in BV-positive HIV infected women. Other causes of genital inflammation could also impact levels of the cationic polypeptides but this was not further evaluated due to their low prevalence in our selected study population (3% were infected with trichomoniasis and only 1 (0.3%) subject was seropositive for syphilis, data not shown).

HIV inhibitory activities of mucosal fluids of low-risk HIV-seronegative individuals have been reported and assigned to a number of different innate molecules in addition to the ones assessed in the present study [3], [11], [25]. Since IgA antibodies from HESN and HIV infected individuals may mediate HIV neutralizing activity [22], these molecules were depleted from all CVS samples prior to measurement of functional activity. In the present study, 37% of the HIV positive, 18% of the HESN and 27% of the low-risk women demonstrated HIV neutralizing activity as assessed in IgA-depleted samples. The high percentage of HIV neutralizing activity seen in the HIV infected group most likely results from the presence of HIV IgG antibodies. Our experimental results showing that depletion of cationic polypeptides abolished the HIV neutralizing activity, and that the functional activity remained in the cationic polypeptide fraction, imply a potential role of these molecules as antiviral agents at local sites. Together, the present results with CVS samples representing Kenyan women at risk of HIV infection extends our previous studies utilizing CVS collected from healthy women from a low HIV endemic area [5]. In the latter study [5], we could restore functional activity by adding back the whole cationic polypeptide fraction to the depleted CVS sample, whereas individual recombinant forms of the most prevalent peptides were not sufficient. In the present study using another HIV neutralizing assay, recombinant LL-37 and HNP1–3, but not SLPI, augmented the functional activity at levels about 10 times that of their physiological concentrations. However, concentrations of recombinant proteins can never be directly compared with that of endogenous molecules.

Additional interactions and synergistic effects of the cationic polypeptides may occur at the mucosal tissue level if the virus penetrates beyond the cervical secretion. Indeed, molecules that perform antimicrobial activity in human secretions may have a counteractive effect in the genital mucosa and contribute to inflammation and recruitment of immune cells that in turn could be infected [26]. Studying the effect of innate immune factors in mucosal fluids in their biological context is essential, since their functions are heavily influenced by the physiological and chemical milieu. Although HNP1–3 and LL37 have antiviral activity *in vitro* they have been associated with an increased rate of HIV acquisition in a cohort of high-risk sex workers [9]. The increased levels of these factors most likely came from ongoing STIs; consequently, the antiviral effect may have been overridden by inflammation and target cell recruitment. Clearly, inflammatory and antiviral properties are delicately balanced during the innate immune response to HIV at mucosal sites.

Several important confounders must be considered in the context of HESN individuals and assessment of mucosal immune responses. Direct effects of sexual intercourse and secondary immune effects of inflammation were here partly controlled for by assessment of PSA and excluding subjects with ongoing STIs. However, hormonal factors and other inflammatory conditions influence the local mucosal environment [2], [27]. Furthermore, other types of assays measuring HIV inhibitory activity or the use of other primary HIV-isolates may reveal important functional activity in addition to the HIV neutralizing activity recorded here. Levels of specific innate immune factors cannot be directly associated with antiviral activity since their biological active form may not be optimally measured by current assays and because they also act in synergy with other innate molecules. Likewise, when the cationic polypeptides where assessed in their recombinant forms, their bioactivity may not exactly reflect the physiological effect of the natural molecule or that of other recombinant variants in previous studies.

Considerable attention has been directed to developing microbicides for topical use to prevent sexual HIV transmission, with promising results from recent clinical trials [28]. Although antiretroviral drugs are the best candidates today, analogues of innate immune molecules that reduce local inflammation and provide an intrinsic HIV inhibitory capacity could be complementary. Characterization of baseline levels of endogenous microbicides in relevant target populations is thus essential, since synergistic activity between endogenous and exogenous microbicide molecules may influence the HIV inhibitory effect. Indeed, study populations representing different geographical regions and with different HIV risk exposures vary in the composition of their normal bacterial microflora, HSV-2 seropositivity and presence of other STIs [29]. Because all these factors contribute to the composition and levels of innate immune molecules, other parameters of mucosal inflammation and ultimately HIV susceptibility warrant examination and confirmation of their beneficial management through pharmaceutical or immunologic means.
